# Granulocyte Colony-Stimulating Factor Protects Mice during Respiratory Virus Infections

**DOI:** 10.1371/journal.pone.0037334

**Published:** 2012-05-16

**Authors:** Tamar Hermesh, Thomas M. Moran, Deepika Jain, Carolina B. López

**Affiliations:** 1 Department of Microbiology and Immunology Institute, Mount Sinai School of Medicine, New York, New York, United States of America; 2 Department of Pathobiology School of Veterinary Medicine and Institute for Immunology, University of Pennsylvania, Philadelphia, Pennsylvania, United States of America; French National Centre for Scientific Research, France

## Abstract

A burst in the production of pro-inflammatory molecules characterizes the beginning of the host response to infection. Cytokines, chemokines, and growth factors work in concert to control pathogen replication and activate innate and adaptive immune responses. Granulocyte colony-stimulating factor (G-CSF) mobilizes and activates hematopoietic cells from the bone marrow, and it has been shown to mediate the generation of effective immunity against bacterial and fungal infections. G-CSF is produced at high levels in the lungs during infection with influenza and parainfluenza viruses, but its role during these infections is unknown. Here we show that during infection of mice with a non-lethal dose of influenza or Sendai virus, G-CSF promotes the accumulation of activated Ly6G^+^ granulocytes that control the extent of the lung pro-inflammatory response. Remarkably, these G-CSF-mediated effects facilitate viral clearance and sustain mouse survival.

## Introduction

Infection of mice with either the murine parainfluenza virus Sendai (SeV) or mouse adapted influenza A virus triggers robust production of pro-inflammatory molecules in the respiratory tract. This cytokine burst is followed by massive infiltration of neutrophils, monocytes, NK cells, DCs and other cell types to the lung. Cytokines and infiltrating cells play a critical role in the clearance of the infection and in the initiation of adaptive immunity [1], [2], [3]. In addition, cytokines produced in the infected lung are transmitted systemically through the blood to alert bone marrow (BM) leukocytes of the presence of the virus, and to condition them to better fight the infection [2].

G-CSF is found in high levels in the lung and blood of mice infected with influenza or parainfluenza virus [2]. G-CSF plays a major role in the mobilization and activation of neutrophils and other myeloid cells from the BM [4], [5] and is currently used to promote mobilization of BM cells in different clinical settings [6], [7], [8]. G-CSF receptor- deficient mice are severely neutropenic and show altered responses to bacterial and fungal infections [9], [10], [11], [12]. However, the role of G-CSF during respiratory viral infection has not been directly evaluated. Here we show that G-CSF regulates lung inflammation during viral infection and that this cytokine is critical for the survival of the host during infection with influenza or the murine parainfluenza virus Sendai.

## Materials and Methods

### Mice and viruses

Age and sex-matched mixed background G-CSF^−/−^ (B6;129P2-*Csf3tm1Ard*/J**)** [9] and control mice of the best approximate mixed background (B6129PF2/J) from The Jackson Laboratory were used in most experiments. G-CSF^−/−^ mice backcrossed to C57BL/6 mice for 7 generations were used where indicated. Mice were bred and housed in pathogen-free conditions and the experiments were performed according to institutionally approved protocols (specific approvals for this project were obtained from the Institutional Animal Care and Use Committees of The Mount Sinai School of Medicine (#08-0160) and The University of Pennsylvania (#803173)). SeV strain 52 and influenza virus strains PR8 (A/PR/8/1934 (H1N1)) and ×31 (A/HK ×31 (H3N2)) were grown in 10-day embryonated chicken eggs (SPAFAS; Charles River Laboratories). Allantoic fluid was snap frozen in an ethanol-dry ice bath and stored at −80°C.

### Mice infection and lung virus titration

Infections with SeV strain 52 were performed in anesthetized mice via intranasal inoculation of 10^4^ infectious viral particles in 35 µl of PBS. This dose was previously determined in our laboratory to reliably establish non-lethal infections [3], [13], [14]. Influenza virus infections were performed via aerosol by placing the mice into a whole body exposure infection chamber (Glass-Col Corp, Model A4212) utilizing a nebulization-infection cycle of 30 min. Influenza virus strain PR8 was aerosolized at a concentration of 10^8^ virus particles in 12 ml PBS. Influenza virus ×31 was aerosolized at a concentration of 10^9^ virus particles in 12 ml PBS. Nebulization was achieved at a vacuum of 30 standard cubic feet per hour (SCFH) and compressed air flow of 15 SCFH. The chamber was UV-decontaminated in between viruses. Under these conditions, 100% of the animals were infected and showed reproducible lung titers in several experiments at every time point analyzed. Virus titers were determined by infecting permissive cell lines LLCMK2 (ATCC; CCL-7.1) (SeV) or MDCK (influenza) [2] with serial dilutions of lung homogenates in the presence 2 µg/ml of trypsin for 72 h at 37°C. Supernatants were tested by hemagglutination of chicken RBCs (Lampire) for the presence of virus particles.

### rhG-CSF injections

250 µg/kg rhG-CSF (Neupogen/Filgrastim, AMGEN, Thousand Oak, CA) [15] or distilled sterile water was injected IP daily to G-CSF^−/−^ mice starting four days before infection. Some mice continued to receive rhG-CSF injections daily for the duration of the experiment.

### *In vivo* neutrophil depletion

Mice were injected intraperitoneally with 400 µg/mouse of an isotype control (2A3) or with rat anti-lymphocyte antigen 6 complex, locus G (Ly6G) antibody (1A8; BioXCell, West Lebanon, NH). Antibody injections were performed at 24 and 0 h prior to infection and every 48 h thereafter.

### Flow cytometry

Lungs were flushed with cold PBS containing 0.5 mM EDTA prior to grinding and digestion with collagenase (Liberase Blendzymes, Roche, Indianapolis, IN). Blood was collected with heparinized capillary tubes. BM was flushed with ice-cold PBS. Erythrocytes were eliminated from the lung, blood, and BM samples using RBCs lysis buffer (BD Biosciences). Single-cell suspensions were incubated with anti-mouse CD16/32 (BD Biosciences) for 10 min at 4°C. The following antibodies from BD Biosciences or eBioscience were used for staining: B220 (RA3-6B2), CD3 (145-2C11), CD4 (GK1.5), CD8α (53-6.7), CD19 (1D3), NK1.1 (PK136), Ter119, CD11b (M1/70), CD11c (HL3), CD45.2 (104), CD115 (AFS98), Ly6C (AL-21), Gr-1 (RB6-8C5) Ly6G (1A8). mPDCA-PE (JF05-1C2.4.1) was obtained from Miltenyi Biotec.

### Cytokine detection in serum and lung

Whole lung was ground in 1.8 ml 0.01% gelatin/PBS. Cytokine concentration was analyzed by multiplex ELISA (Milipore). G-CSF concentration was measured by ELISA (R&D).

### Detection of virus specific antibodies

ELISA plates were coated with purified SeV (5 µg/ml) overnight. Plates were blocked with PBS/BSA for 2 h. Dilutions of lung homogenate from infected and non-infected mice were incubated overnight at 4°C. SeV specific antibodies of different isotypes were detected by peroxidase-conjugated goat anti-mouse antibodies (Jackson ImmunoResearch).

### In vivo cytotoxic T lymphocyte (CTL) assay

Splenocytes from naive mice were pulsed with 20 µg/ml SeV NP_324–332_ peptide or with 20 µg/ml influenza PR8 NP_(366–374)_ peptide in PBS as previously described [14].

### *In vivo* phagocytosis assay

Yellow-green fluorescent latex particles (10 µm, Polysciences) or unlabeled particles were diluted 1∶20 in PBS and 30 µl of the dilution was administered intranasally to anesthetized mice. Mice were sacrificed 4 h later and bead uptake by lung leukocytes was analyzed by flow cytometry.

### Histology and bronchoalveolar lavage (BAL) analysis

BAL was obtained after one cycle of infusion and aspiration into the lungs of 1 ml of sterile saline as previously described [16]. Cell pellets obtained after BAL centrifugation at 400 g were suspended in 500 µl of PBS and counted manually for total cell counts (TLC). Cell-free BAL was aliquoted and frozen until further analysis. Total protein in BAL was measured by the Coomasie blue spectrophotometric assay (Bio Rad) using BSA dilutions as standards. After lavage, the left lobe of the lung was inflation-fixed with 0.5 ml of 10% neutral buffered formalin for histological analysis. Paraffin sections from fixed lungs were stained with hematoxylin and eosin and scored on the basis of presence and absence of peribronchiolar and alveolar infiltration, vascular congestion and alveolar edema [17].

### Enrichment of Ly6G^+^ cells from bone marrow

Bone marrow from femur and tibia of WT and G-CSF^−/−^ mice was flushed out with serum-free DMEM (Invitrogen) supplemented with antibiotics. Ly6G^+^ cells were enriched by positive selection using magnetic beads. Non-specific antibody binding on cells was blocked using anti-CD16/CD32. Up to 1×10^8^ bone marrow cells were incubated on ice for 15 min with biotin-anti Ly6G antibody (1A8, Biolegend), followed by incubation with anti-biotin microbeads (Miltenyi Biotech). An enrichment column was used per manufacturer's instructions to collect Ly6G^+^ and Ly6G^−^ fractions. The separated fractions were spun at 400 g for 10 min and the cell pellets suspended in Trizol (Invitrogen) for RNA extraction and qPCR analysis. A sample of each sample was analyzed by cytospin, confirming a >95% enrichment of polymorphonuclear cells in the Ly6G^+^ fraction (not shown).

### Quantitative RT-PCR

RNA was extracted from BMDCs, lung fibroblasts, and RAW 264.7 cells at the indicated time points after virus infection using TRIzol (Invitrogen Life Technologies) or the High Pure RNA Isolation Kits (Roche). RNA was measured and equivalent amounts of RNA from each sample (0.5–1.0 µg) were reverse transcribed using the High Capacity RNA-to cDNA Kit (Applied Biosystems). cDNA was diluted to a concentration of 10 µg/µl min distilled water, and PCR reactions were performed in triplicate using specific primers and the Power SYBR® Green PCR Master Mixture (Applied Biosystems). Normalization was conducted based on levels of α-tubulin and rps11. The following primer sequences were used: α-*tub: for-* 5′TGCCTTTGTGCACTGGTATG3′ *rev*-5′CTGGAGCAGTTTGACGACAC3′, *rps11: for-* 5′CGTGACGAAGATGAAGATGC3′ *rev-* 5′GCACATTGAATCGCACAGTC3′, *ela-2: for-* 5′TGGAGGTCATTTCTGTGGTG3′ *rev-* 5′CTGCACTGACCGGAAATTTAG3′, *mpo: for-* 5′ CAAGGCCTTTCAATGTTACAGA3′ *rev-* 5′TGTCACCCTCACGTCCTG3′, *cebp-beta: for-* 5′ATCGACTTCAGCCCCTACCT3′ *rev-* 5′TAGTCGTCGGCGAAGAGG3′, *Il-10: for-* 5′TGTCCAGCTGGTCCTTTGTT3′ *rev-* 5′ACTGCACCCACTTCCCAGT3′, *ccl-5: for-* 5′GCAGCAAGTGCTCCAATCTT3′ *rev-* 5′CAGGGAAGCGTATACAGGGT3′ (applied biosystem, Viia7).

### Statistical analysis

Results are expressed as means ± s.d. Statistical significance was determined by two-tailed Student's *t* test unless otherwise indicated. Values of at least *p*<0.05 were considered significant.

## Results

### G-CSF produced during infection with SeV or influenza virus promotes host survival and viral clearance

**Figure 1:**
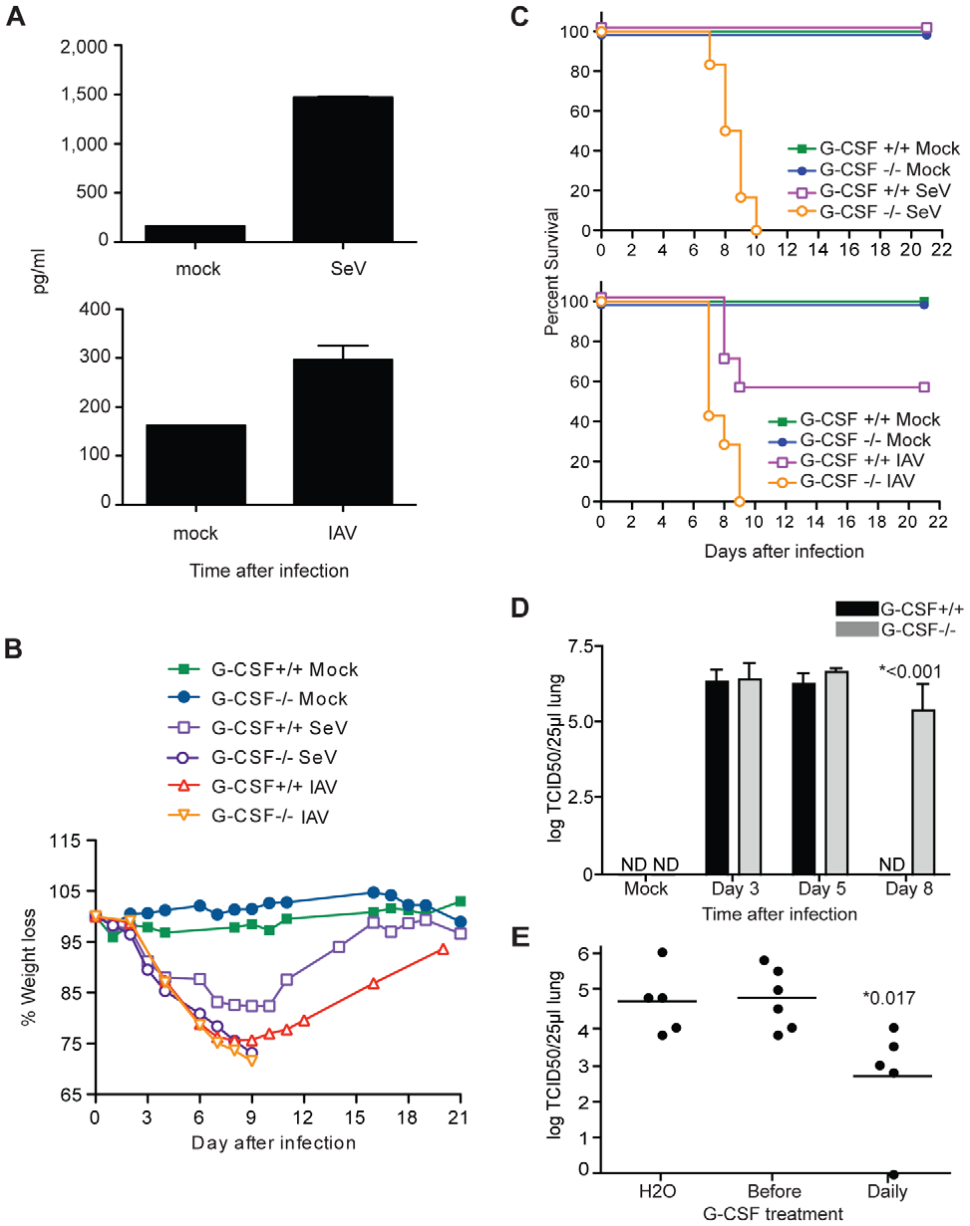
Vital role for G-CSF produced in the lung of infected mice. (A) G-CSF measured in the sera of mice 3 days after infection with SeV or with influenza virus ×31 (IAV). (B) Percent weight change of G-CSF^+/+^ and G-CSF^−/−^ mice infected with SeV (n = 6), influenza ×31 (n = 7) or mock treated controls. *P*>0.6 two-way ANOVA for infected groups. (C) Survival of G-CSF^+/+^ and G-CSF^−/−^ mice monitored after infection with SeV (n = 6, *p* = 0.0009, logrank test) or influenza ×31 (n = 7, *p* = 0.012, logrank test). (D) SeV titer in the lungs of G-CSF^+/+^ and G-CSF^−/−^ mice at the indicated time points (n = 8). (E) Viral titers in the lungs of G-CSF^−/−^ mice infected with SeV and treated with daily injections of H_2_O or rhG-CSF four times before infection (Before) or continued treatment throughout the course of the experiment (Daily). ND: non-detected, NM: non-measured. Asterisks indicate *p* values. Data presented is representative of more than two independent experiments.

As reported earlier [2], high levels of G-CSF can be detected in the lung and serum of mice infected with influenza or SeV (Figure 1A). To assess the role of G-CSF during respiratory virus infection, mixed background G-CSF^+/+^ and G-CSF^−/−^ mice were infected with mouse adapted influenza virus or with SeV. G-CSF^+/+^ and G-CSF^−/−^ infected mice lost weight at a similar rate after infection regardless of the virus (Figure 1B); strikingly, in the absence of G-CSF all mice succumbed to the infection between days 7 and 10 after infection (Figure 1C). These data highlight an essential role for G-CSF in protecting the host from death during infection with normally non-lethal respiratory viruses.

To further characterize the protective role of G-CSF during respiratory viral infection, we focused on infections with SeV, a natural mouse pathogen. To define whether G-CSF affects viral load, we compared viral titers in the lungs of infected G-CSF^+/+^ and G-CSF^−/−^ mice. SeV replicated equally well in the lungs of G-CSF^+/+^ and G-CSF^−/−^ mice in the days that follow infection. However, while G-CSF^+/+^ mice cleared SeV by day eight-post infection, virus was still found in the lungs of G-CSF^−/−^ at this time point (Figure 1D). Administration of daily doses of recombinant human G-CSF (rhG-CSF) throughout the course of infection improved the ability of G-CSF^−/−^ mice to control virus replication (Figure 1E), confirming an essential role of G-CSF for efficient viral clearance.

### G-CSF deficient mice show increased production of pro-inflammatory molecules in the lung during infection

**Figure 2:**
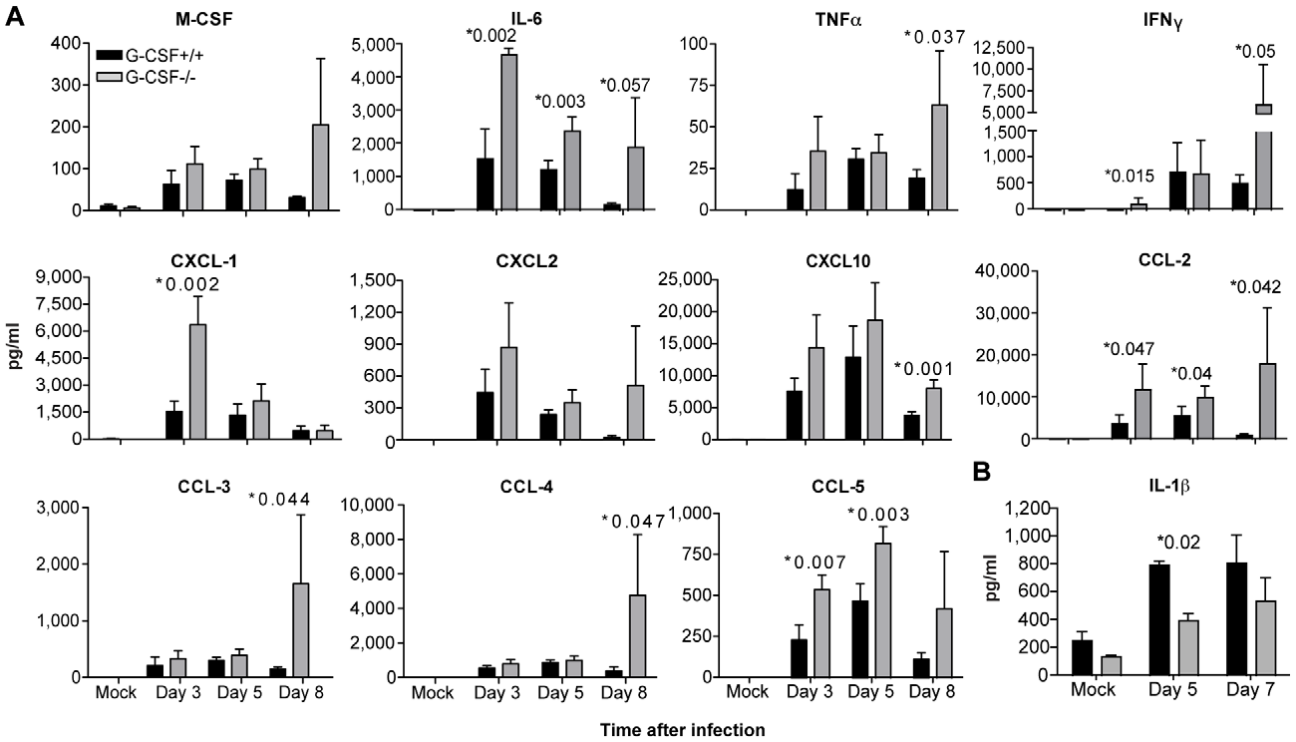
G-CSF regulates the lung inflammatory response during virus infection. (A–B) Cytokine secretion in the lungs of G-CSF^−/−^ and G-CSF^+/+^ mice infected with SeV was measured by multiplex ELISA (n = 4).

To determine whether the lung anti-viral response was altered in G-CSF^−/−^ mice, we profiled the production of pro-inflammatory cytokines in the lungs at different times after infection with SeV (Figures 2A). G-CSF^−/−^ mice showed higher levels of pro-inflammatory molecules including cytokines (IL-6, TNF, IFNγ) and chemokines (CCL2, CXCL1, CCL5) compared with control mice. The differences in pro-inflammatory molecules were most evident at day 8 post-infection correlating with the sustained presence of virus in G-CSF^−/−^ mice at this time point (Figure 1D). Remarkably, in contrast to all other cytokines analyzed, the mRNA and protein levels of IL-1β were significantly reduced in G-CSF^−/−^ mice at day 5 post-infection (Figure 2B and data not shown).

### G-CSF deficient mice develop adaptive immune responses against the virus

**Figure 3:**
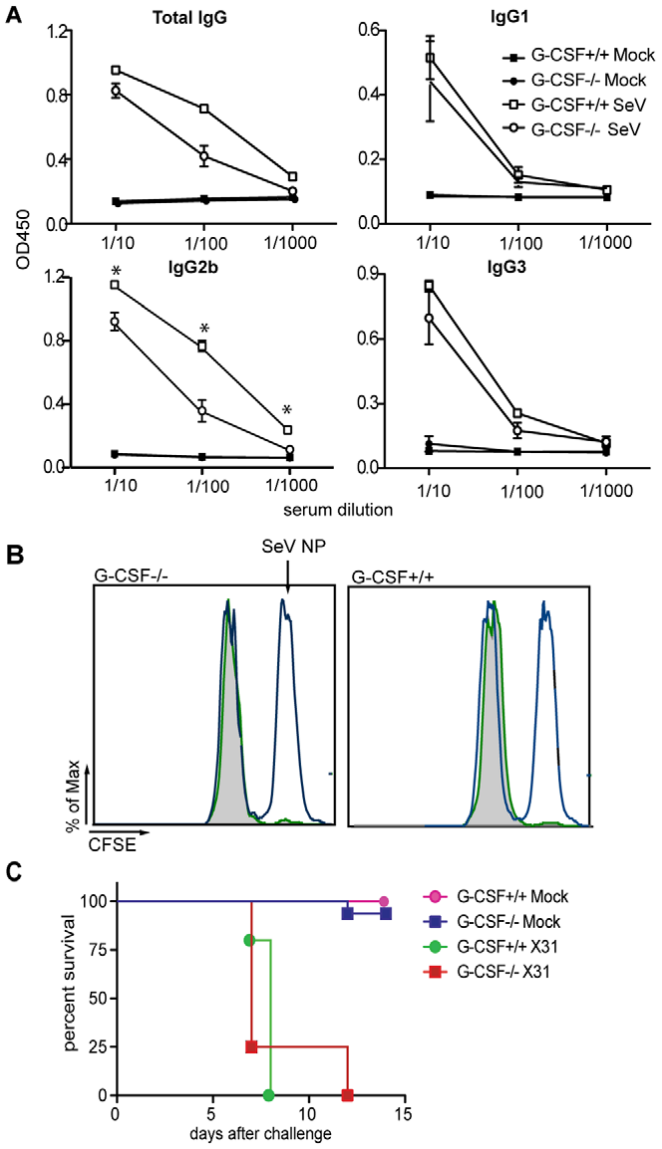
Adaptive immunity in the absence of G-CSF. (A) SeV specific antibodies in lung homogenate of G-CSF^+/+^ and G-CSF^−/−^ mice measured by ELISA 7 days after infection (n = 4). Experiment shown is representative of two independent experiments. IgG2a *p*>0.15, IgG3 *p*>0.07, IgG2b *p*<0.006, total IgG *p*<0.03 between infected G-CSF^+/+^ and infected G-CSF^−/−^ for all dilutions. (B) Mock and SeV infected G-CSF^+/+^ or G-CSF^−/−^ mice were injected i.v. 7 days after infection with CFSE-labeled peptide-pulsed splenocytes. Splenocytes labeled with a high dose of CFSE were pulsed with SeV NP peptide and those labeled with a low dose of CFSE were pulsed with the irrelevant influenza virus (PR8) NP peptide. Empty histograms show CFSE staining on mock-infected animals 24 h after injection of labeled/pulsed splenocytes. Filled histograms correspond to CFSE staining on SeV infected mice 24 h after injection of labeled/pulsed splenocytes (n = 4 per group). Experiment shown is representative of two independent experiments. (C) Survival of G-CSF^+/+^ and G-CSF^−/−^ mice infected with a sublethal dose of influenza virus ×31 or mock treated and challenged at day 19 with a normally lethal dose of influenza virus PR8. Survival was monitored daily (n = 14/16 mice in ×31 challenged groups, n = 5 in mock treated controls). Experiment shown is representative of two independent experiments.

We next evaluated the effect of G-CSF on the development of adaptive immunity against the virus. G-CSF deficient mice generated SeV specific antibodies, although anti-viral antibodies of the IgG2b isotype were significantly reduced (Figure 3A). The absence of G-CSF did not affect the development of effector cytotoxic T lymphocytes (CTLs) as evidenced by efficient killing of specific target cells *in vivo* (Figure 3B). To evaluate whether G-CSF deficient mice developed protective cellular immunity, we utilized a well-characterized model of heterosubtypic infection with influenza virus [18]. G-CSF^−/−^ mice infected with a sub-lethal dose of influenza strain ×31 (H3N2) were protected against challenge with a normally lethal dose of the heterosubtypic influenza strain PR8 (H1N1) (Figure 3C) that is not susceptible to neutralization by anti-X31 antibodies. These data indicate that the presence of G-CSF determines the quality of the initial inflammatory response to respiratory viruses but has minimal effects on the development of anti-viral adaptive immune responses.

### G-CSF deficient mice show reduced cellular infiltrate and tissue damage during infection

**Figure 4:**
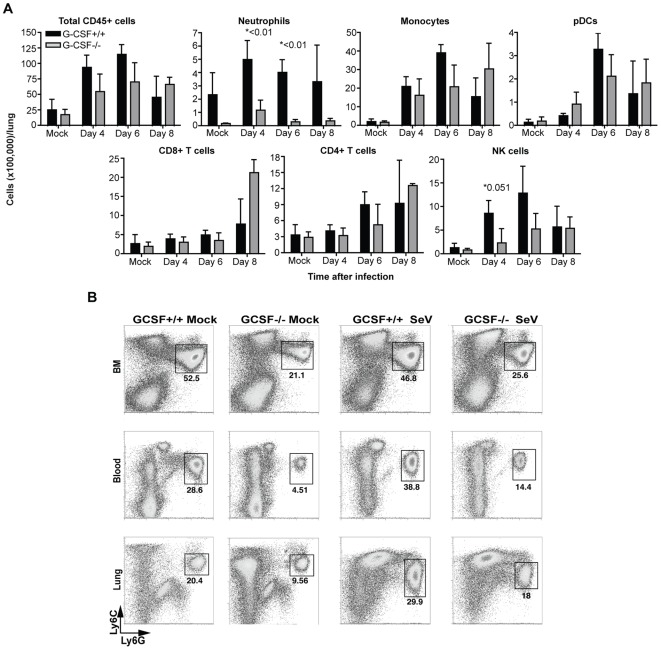
G-CSF regulates the cellular infiltrate during virus infection. (A) Cell numbers in the lungs of SeV infected mice calculated at the indicated time points from total cell counts and flow cytometry analysis. Populations were identified with the following markers: neutrophils (CD11b^+^Ly6c^+^Ly6G^+^), monocytes (CD11b^+^Ly6C^+^Ly6G^−^), pDCs (CD11b^−^B220^+^mPDCA^+^), CD4^+^ T cells (CD3^+^CD4^+^CD8^−^), CD8^+^ T cells (CD3^+^CD8^+^CD4^−^), and NK cells (CD11b^+^NK1.1^+^). Pre-gated on PI^−^CD45^+^ cells. Error bars represent standard deviation from the mean. Asterisks indicate *p* values (n = 3). (B) Ly6G^+^ granulocytes in the lung, BM and blood of G-CSF^−/−^ and G-CSF^+/+^ mice mock treated or infected with SeV for 4 days. Pre-gated on PI^−^CD45^+^CD11b^+^ cells. Results are representative of more than two independent experiments.

We next analyzed the composition of the lung cellular infiltrate during infection. The numbers of infiltrating monocytes and plasmacytoid DCs in the lung of mock and infected mice were not significantly different in the absence or presence of G-CSF (Figures 4A). NK cells were transiently reduced in the lung of G-CSF^−/−^ mice at early times after infection but this trend did not reach statistical significance. Notably, CD8^+^ T cells were elevated on day 8 after infection in G-CSF^−/−^ mice, possibly in response to higher virus titers compared to controls at this time point. As expected [9], mock-infected G-CSF^−/−^ mice had lower levels of Ly6G^+^ granulocytes in the BM, blood, and lung compared to G-CSF^+/+^ mice. Despite an increase in the number of granulocytes in the lung during infection with SeV, these cells were significantly lower in the lung of G-CSF^−/−^ mice compared to controls at all time points analyzed (Figures 4A and B).

**Figure 5:**
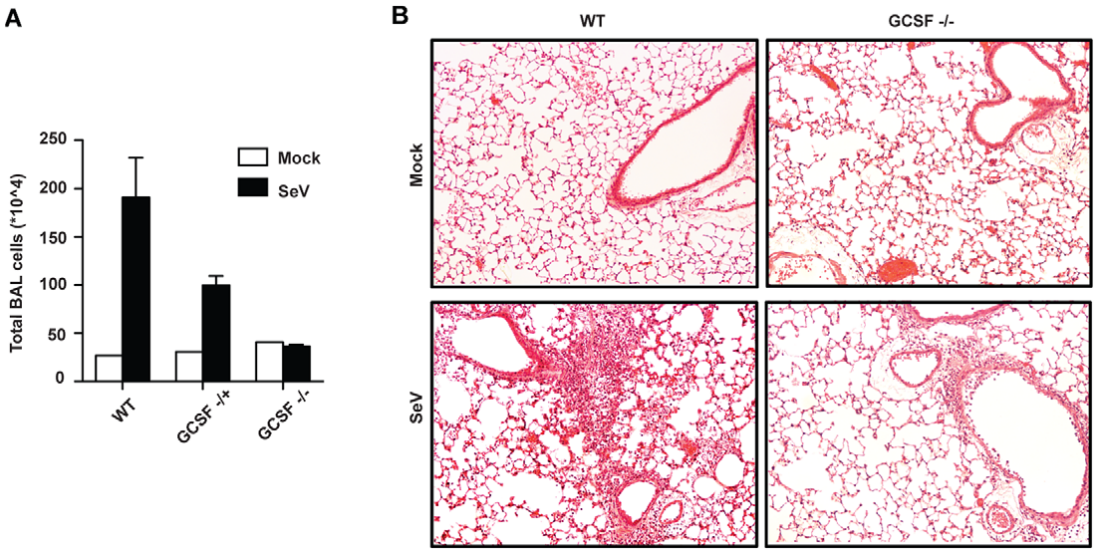
G-CSF deficiency reduces lung infiltrate during virus infection. (A) Total cell counts in cytospins made with bronchoalveolar lavage (BAL) 8 days after infection with SeV. (B) Representative pictures of H&E-stained lung sections from naive and SeV infected G-CSF^+/+^ and G-CSF^−/−^ C57BL/6 mice.

Reduced cellular infiltration to the lung of G-CSF^−/−^ mice was also apparent in the analysis of the BAL of infected mice. Total lymphocytes (TLC) in the BAL were significantly reduced in infected G-CSF^−/−^ and G-CSF^+/−^ mice compared to infected G-CSF^+/+^ controls (Figure 5A). Histological analysis of the lung showed marked peribronchiolar and alveolar infiltration of cells post-infection in WT mice, which was significantly attenuated in similarly infected G-CSF^−/−^ mice (Figure 5B). However, 25–30% higher levels of total protein were observed in BAL of G-CSF^−/−^ mice after infection (963 µg/ml in WT vs. 1207 µg/ml in G-CSF^−/−^ mice; data not shown), suggesting partial loss of bronchoalveolar-epithelial integrity in mice deficient in G-CSF. Importantly, the experiments in Figure 5 were performed in G-CSF^−/−^ mice backcrossed to the C57BL/6 background, demonstrating that the protective phenotype observed in mice with mixed genetic background is maintained in the more resistant C57BL/6 mouse strain.

### Reduced numbers of activated Ly6G^+^ granulocytes are present in the lungs of G-CSF deficient mice

**Figure 6:**
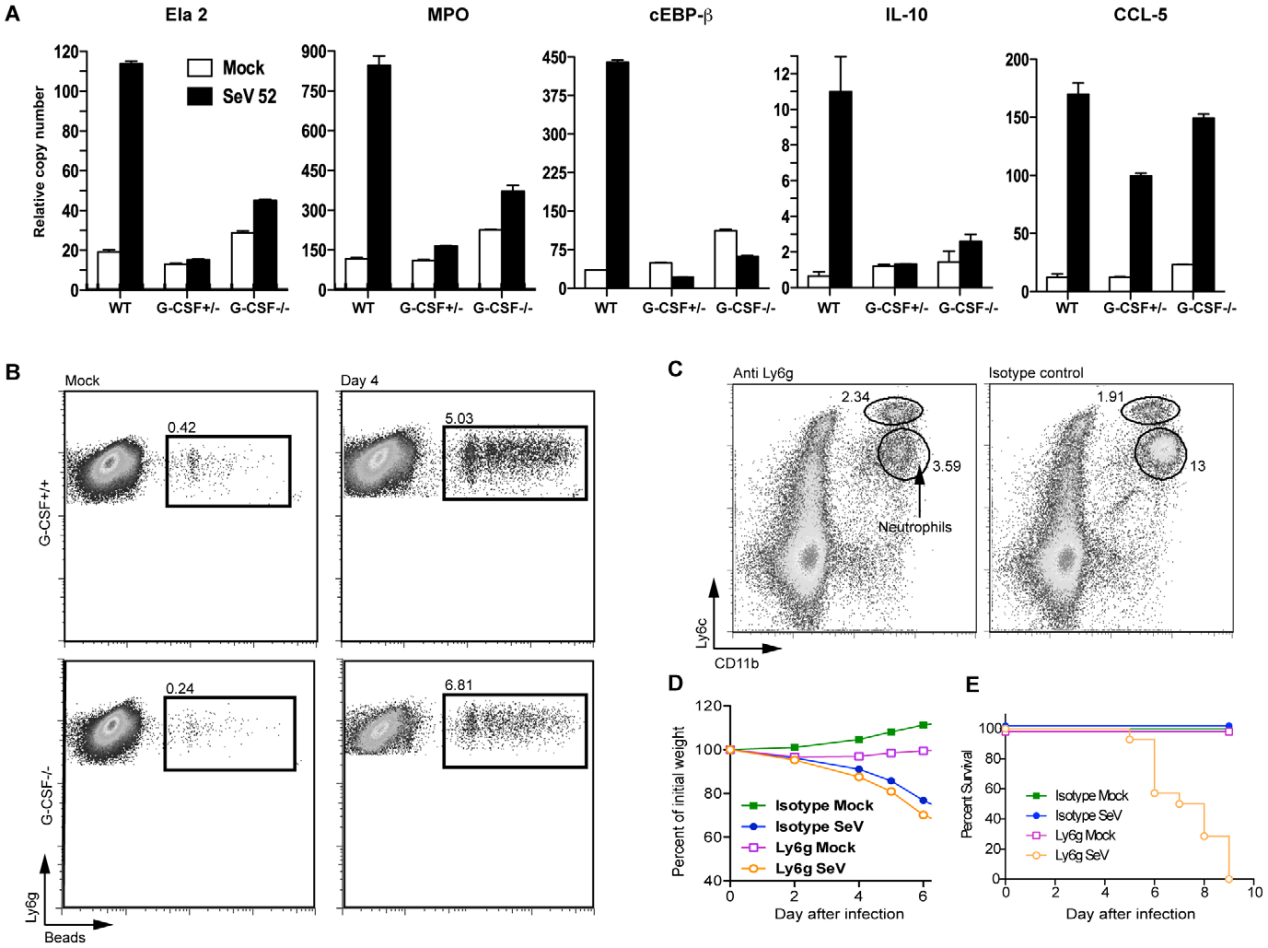
Activated Ly6G^+^ granulocytes protect from mortality during virus infection. (A) Cytokine gene expression in Ly6G^+^ enriched bone marrow cells. (B) Uptake of fluorescent latex beads by neutrophils (PI^−^CD45^+^Ly6G^+^CD11b^+^) in the lung of GCSF^+/+^ and G-CSF^−/−^ mice four days after infection with SeV (n = 4). (C) Representative flow cytometry analysis of neutrophils (CD11b^+^Ly6c^int^) and monocytes (CD11b^+^Ly6c^hi^) in the blood of mice injected with a single dose of anti-Ly6G antibody (1A8) or an isotype control. Neutrophil depleted WT C57BL6 mice were infected with SeV and monitored for (D) weight loss (*p*>0.9 two way ANOVA for infected groups) and (E) survival (n = 10 for isotype treated, SeV infected and n = 20 for Ly6G depleted and SeV infected. Logrank test, *p*<0.0001). Experiments shown are representative of two or three independent experiments.

To evaluate the effect of G-CSF in the activation status of Ly6G^+^ granulocytes during virus infection, we analyzed the expression of genes associated with neutrophil activation in Ly6G^+^ granulocytes of G-CSF^−/−^, G-CSF^+/−^, and G-CSF^+/+^ mice 4 days after infection. To avoid potential confounding effects of direct virus infection of granulocytes in the lung, we analyzed granulocytes residing in the distal bone marrow of the infected mice, as we have shown that these cells respond to cytokines produced by the lung during infection [2]. As shown in Figure 6A, G-CSF induced the expression of a number of genes associated with neutrophil activation (elastase, Ela-2; myeloperoxidase, MPO and C/EBPβ) in bone marrow Ly6G^+^ cells of WT mice. Unexpectedly, the regulatory cytokine IL-10 was also expressed at high levels in Ly6G^+^ cells from infected WT mice. Expression of all these genes was severely impaired in Ly6G^+^ cells from G-CSF^−/−^ and G-CSF^+/−^ mice, suggesting a critical role of G-CSF in generating a functionally active population of Ly6G^+^ granulocytes during virus infection. Expression of the unrelated chemokine CCL5 was not different in WT and G-CSF^−/−^ suggesting that the hyporesponsiveness in SeV-infected Ly6G^+^ cells is restricted to granulocyte-specific genes.

We next examined whether the phagocytic ability of neutrophils recruited to the lung during infection was altered in G-CSF^−/−^ mice. To test this, G-CSF^−/−^ and G-CSF^+/+^ mice were infected with SeV and four days later fluorescently labeled latex beads were administered intranasally [19]. We measured the efficiency of bead phagocytosis by granulocytes using flow cytometry. Figure 6B shows that despite a severe reduction in the number of phagocytic granulocytes in the lung of G-CSF^−/−^ mice, there is an equal percentage of bead positive cells within granulocytic populations of G-CSF^−/−^ and G-CSF^+/+^ mice. These data indicate that the virus-activated phagocytic ability of granulocytes is unaffected by the presence or absence of G-CSF, although the number of activated phagocytic cells present in the lung of G-CSF^−/−^ mice is significantly reduced.

To test whether a reduction of the number of granulocytes recapitulates the enhanced pathology observed in the absence of G-CSF, we used an anti-Ly6G antibody to deplete Ly6G^+^ cells from WT mice [20], [21]. Granulocyte counts were reduced by 70% in the blood of WT mice twenty-four hours after a single dose of anti-Ly6G antibody (Figure 6C) mimicking the percent reduction of granulocytes in the blood of G-CSF^−/−^ mice compared to WT controls (Figure 4B). Similar to G-CSF^−/−^ mice, depletion of Ly6G^+^ cells from WT mice resulted in a weight loss comparable to that of control mice (Figure 6D), but also resulted in enhanced mortality post-infection (Figure 6E).

Together with the observed dramatic reduction in the expression of functional genes, these data suggest a critical role for activated Ly6G^+^ granulocytes in the G-CSF-mediated protection from death during respiratory viral infections.

## Discussion

We have shown that the absence of G-CSF results in decreased survival of the host during a respiratory tract viral infection (Figure 1) independently of the generation of cellular and humoral adaptive immunity (Figure 3). G-CSF regulates mobilization of granulocytes, hematopoietic stem cells, and other myeloid cells from the BM [5], [22] and plays a critical role in the activation of neutrophils that control bacterial and fungal infections [9], [10], [11], [12]. In the present work we demonstrated that G-CSF regulates the outcome of respiratory viral infections by stimulating the mobilization and recruitment of large numbers of activated granulocytes to the infected lung, thereby promoting viral clearance and eliciting a distinct survival advantage.

Neutrophils have been shown to control lung pathology through the release of suppressor cytokines such as IL-10 [23] and it is possible that this granulocyte subset contributes to host survival during virus infection by suppressing the cytokine storm induced in the lung in response to infection. Our data show that Ly6G^+^ granulocytes in the bone marrow of infected WT mice express high levels of IL-10 in response to pro-inflammatory signals. This response is abrogated in the absence of G-CSF, suggesting a possible mechanism for the G-CSF-dependent suppressor activity.

Using an antibody-mediated neutrophil depletion model, a series of recent reports have suggested a role for neutrophils during infection with influenza virus [24], [25], [26], [27], [28]. G-CSF-deficient mice provide a complementary method for the reduction of functional granulocytes, confirming a critical role for these cells in controlling lung pathology during infection with normally non-lethal respiratory viruses. Interestingly, the effect of neutrophils in lung pathology is influenced by the virus strain used (23), suggesting that additional factors contribute to the overall effect of granulocyte-mediated protection against viral infection.

Currently, G-CSF is used in the clinic for the treatment of BM transplant recipients to aid in their recovery and to protect them from bacterial infections. It may be beneficial to take a closer look at individuals that suffer from severe virus infections and examine the status of their neutrophil population. Is possible that during certain infections, or under specific health conditions, G-CSF treatment may promote quick virus clearance and recovery from infection.

## References

[1] Kohlmeier JE, Woodland DL (2009). Immunity to respiratory viruses.. Annu Rev Immunol.

[2] Hermesh T, Moltedo B, Moran TM, Lopez CB (2010). Antiviral instruction of bone marrow leukocytes during respiratory viral infections.. Cell Host Microbe.

[3] Moltedo B, Lopez CB, Pazos M, Becker MI, Hermesh T (2009). Cutting edge: stealth influenza virus replication precedes the initiation of adaptive immunity.. J Immunol.

[4] Semerad CL, Liu F, Gregory AD, Stumpf K, Link DC (2002). G-CSF is an essential regulator of neutrophil trafficking from the bone marrow to the blood.. Immunity.

[5] Basu S, Hodgson G, Katz M, Dunn AR (2002). Evaluation of role of G-CSF in the production, survival, and release of neutrophils from bone marrow into circulation.. Blood.

[6] Cashen AF, Lazarus HM, Devine SM (2007). Mobilizing stem cells from normal donors: is it possible to improve upon G-CSF?. Bone marrow transplantation.

[7] Pamphilon D, Nacheva E, Navarrete C, Madrigal A, Goldman J (2008). The use of granulocyte-colony-stimulating factor in volunteer unrelated hemopoietic stem cell donors.. Transfusion.

[8] Anderlini P, Champlin RE (2008). Biologic and molecular effects of granulocyte colony-stimulating factor in healthy individuals: recent findings and current challenges.. Blood.

[9] Lieschke GJ, Grail D, Hodgson G, Metcalf D, Stanley E (1994). Mice lacking granulocyte colony-stimulating factor have chronic neutropenia, granulocyte and macrophage progenitor cell deficiency, and impaired neutrophil mobilization.. Blood.

[10] Liu F, Wu HY, Wesselschmidt R, Kornaga T, Link DC (1996). Impaired production and increased apoptosis of neutrophils in granulocyte colony-stimulating factor receptor-deficient mice.. Immunity.

[11] Basu S, Quilici C, Zhang HH, Grail D, Dunn AR (2008). Mice lacking both G-CSF and IL-6 are more susceptible to Candida albicans infection: critical role of neutrophils in defense against Candida albicans.. Growth Factors.

[12] Basu S, Hodgson G, Zhang HH, Katz M, Quilici C (2000). “Emergency” granulopoiesis in G-CSF-deficient mice in response to Candida albicans infection.. Blood.

[13] Brimnes MK, Bonifaz L, Steinman RM, Moran TM (2003). Influenza virus-induced dendritic cell maturation is associated with the induction of strong T cell immunity to a coadministered, normally nonimmunogenic protein.. J Exp Med.

[14] Lopez CB, Yount JS, Hermesh T, Moran TM (2006). Sendai virus infection induces efficient adaptive immunity independently of type I interferons.. J Virol.

[15] Tamura M, Hattori K, Nomura H, Oheda M, Kubota N (1987). Induction of neutrophilic granulocytosis in mice by administration of purified human native granulocyte colony-stimulating factor (G-CSF).. Biochem Biophys Res Commun.

[16] Jain D, Atochina-Vasserman EN, Tomer Y, Kadire H, Beers MF (2008). Surfactant protein D protects against acute hyperoxic lung injury.. American journal of respiratory and critical care medicine.

[17] Jain D, Atochina-Vasserman E, Kadire H, Tomer Y, Inch A (2007). SP-D-deficient mice are resistant to hyperoxia.. American journal of physiology Lung cellular and molecular physiology.

[18] Lopez CB, Fernandez-Sesma A, Czelusniak SM, Schulman JL, Moran TM (2000). A mouse model for immunization with ex vivo virus-infected dendritic cells.. Cell Immunol.

[19] Jakubzick C, Helft J, Kaplan TJ, Randolph GJ (2008). Optimization of methods to study pulmonary dendritic cell migration reveals distinct capacities of DC subsets to acquire soluble versus particulate antigen.. Journal of Immunological Methods.

[20] Daley JM, Thomay AA, Connolly MD, Reichner JS, Albina JE (2007). Use of Ly6G-specific monoclonal antibody to deplete neutrophils in mice.. J Leukoc Biol.

[21] Tate MD, Deng Y-M, Jones JE, Anderson GP, Brooks AG (2009). Neutrophils Ameliorate Lung Injury and the Development of Severe Disease during Influenza Infection.. J Immunol.

[22] Greenbaum AM, Link DC (2011). Mechanisms of G-CSF-mediated hematopoietic stem and progenitor mobilization.. Leukemia.

[23] Zhang X, Majlessi L, Deriaud E, Leclerc C, Lo-Man R (2009). Coactivation of Syk kinase and MyD88 adaptor protein pathways by bacteria promotes regulatory properties of neutrophils.. Immunity.

[24] Narasaraju T, Yang E, Samy RP, Ng HH, Poh WP (2011). Excessive neutrophils and neutrophil extracellular traps contribute to acute lung injury of influenza pneumonitis.. Am J Pathol.

[25] Hashimoto Y, Moki T, Takizawa T, Shiratsuchi A, Nakanishi Y (2007). Evidence for Phagocytosis of Influenza Virus-Infected, Apoptotic Cells by Neutrophils and Macrophages in Mice.. J Immunol.

[26] Tate MD, Ioannidis LJ, Croker B, Brown LE, Brooks AG (2011). The role of neutrophils during mild and severe influenza virus infections of mice.. PloS one.

[27] Crowe CR, Chen K, Pociask DA, Alcorn JF, Krivich C (2009). Critical role of IL-17RA in immunopathology of influenza infection.. J Immunol.

[28] Akk AM, Simmons PM, Chan HW, Agapov E, Holtzman MJ (2008). Dipeptidyl peptidase I-dependent neutrophil recruitment modulates the inflammatory response to Sendai virus infection.. J Immunol.

